# Synergistic effects of cryoablation and GM-CSF in colorectal liver metastases management in tumor-bearing mice

**DOI:** 10.22038/ijbms.2025.82910.17980

**Published:** 2025

**Authors:** Junfeng Wang, Dalu Kong

**Affiliations:** 1 Department of Colorectal Cancer, Tianjin Medical University Cancer Institute and Hospital, Tianjin, 300060, China; 2 National Clinical Research Center for Cancer, Tianjin, 300060, China; 3 Tianjin Key Laboratory of Cancer Prevention and Therapy, Tianjin, 300060, China; 4 Tianjin’s Clinical Research Center for Cancer, Tianjin, 300060, China

**Keywords:** Colorectal neoplasms, Cryosurgery, Cytokines, Dendritic cells, Granulocyte-macrophage - colony-stimulating factor, Immunity, Liver neoplasms, Tumor microenvironment

## Abstract

**Objective(s):**

The use of cryoablation for colorectal liver metastases (CLM) remains limited and controversial. This study aimed to investigate the antitumor immune response following cryoablation combined with granulocyte-macrophage colony-stimulating factor (GM-CSF) treatment in a CLM mouse model.

**Materials and Methods:**

A CLM mouse model was established using BALB/c mice. The tumor-bearing mice were randomly divided into Control group, GM-CSF group, cryoablation group, and cryoablation + GM-CSF group. Tumor size, survival time, dendritic cells (DCs) count, serum cytokine levels (IL-4, IFN-γ), and the Th1/Th2 ratio (IFN-γ/IL-4) were compared among the four groups.

**Results:**

The combination of cryoablation and GM-CSF demonstrated synergistic effects, resulting in the smallest tumor lesion, longest mean survival time, and highest DC count on day 21 post-treatment compared to other groups. Both cryoablation alone and combined with GM-CSF significantly increased serum IFN-γ levels and suppressed IL-4 levels on day 21 compared to pre-treatment levels (*P*<0.05). Notably, the combination of cryoablation and GM-CSF significantly elevated the Th1/Th2 ratio (*P*<0.05).

**Conclusion:**

Combining cryoablation with GM-CSF treatment holds promise for CLM management. It exhibits increased DC infiltration within the tumor microenvironment, enhanced immune responses, and prolonged survival in tumor-bearing mice.

## Introduction

Colorectal cancer, a leading cause of cancer-related death, poses a significant global health challenge (1). The rising incidence of this disease places a heavy burden on patients and their families (1, 2). Liver metastasis is a common and often fatal complication, with approximately 50% of colorectal cancer patients developing colorectal liver metastases (CLM) and 30% succumbing to the disease with the liver as the only metastatic site (3). While liver resection offers a potential cure, only 10-15% of CLM patients are eligible for this procedure due to comorbidities and age (4). Despite the potential benefits of neoadjuvant chemotherapy, its use worldwide faces unique challenges, especially in China (5). Therefore, exploring alternative therapies for CLM is crucial to improve patient outcomes. Such innovative treatments may hold promise for overcoming the limitations of current options and providing hope for a more effective and personalized approach to this complex disease. 

The evidence regarding the use of cryoablation for CLM remains limited and conflicting. Although cryoablation was among the initial thermal ablation techniques utilized in tumor management, its application has been limited due to concerns regarding a high local recurrence rate, elevated complication rates, and the emergence of alternative novel local ablation therapies** (**6**)**. Conflicting evidence regarding the efficiency of cryoablation has been reported. Ng *et al.* revealed that hepatic cryotherapy could be a safe and effective treatment for CLM, even in cases of unresectable disease (7). Similarly, a study found that hepatic cryotherapy was a highly flexible, well-visualized, and safe procedure with very low local recurrence rates in tumor lesions near central biliary structures or adjacent critical anatomical structures (8). Conversely, Seifert JK and Morris DL observed a high risk of perioperative deaths associated with cryo-shock following cryoablation (9). In a review, a high local recurrence rate of 12–39% was reported across 26 cryoablation-related studies (10). Furthermore, some studies indicated that cryoablation alone was insufficient to induce abscopal immune-regulatory effects (11, 12). Cryoablation combined with irreversible electroporation has shown promising short- to mid-term results in liver tumor treatment, achieving high local tumor control within 6 months. However, long-term data still needed to be included (13). Given its notable immune interactions (14), the clinical impact of cryoablation may have been underestimated.

In various biological processes, GM-CSF plays a crucial role in promoting the generation, differentiation, and function of granulocytes and macrophages and regulating immune responses and inflammatory processes (15, 16). GM-CSF could function as an immunological adjuvant and significantly enhance the efficiency of antitumor immune responses by promoting the maturation and activation of DCs and enhancing the function of antigen-presenting cells (17). GM-CSF, acting as an adjuvant, may also enhance the efficacy of colorectal cancer and CLM treatments (18, 19).

Here, we assumed that cryoablation combined with GM-CSF could play a role in CLM treatment by enhancing antitumor immune response. In this study, a mouse model of CLM was established. The survival time and the changes of tumor size, DC counts in tumor tissue, serum cytokine levels (IFN-γ and IL-4), and Th1/Th2 ratio were compared among Control group (CLM mice given sham treatment), Group A (CLM mice given mouse GM-CSF injection of 250 μg/day for three consecutive days), Group B (CLM mice given cryoablation treatment), and Group C (CLM mice given combined cryoablation and GM-CSF treatment). 

## Materials and Methods

### Animal and cell culture

Ninety male BALB/c mice (6–8 weeks old, weighing 18–22 g) were purchased and housed in a specific pathogen-free (SPF) environment at 22 ± 2 °C with a 12-hour light/dark cycle. Mice had free access to food and water. The CT-26 mouse colon cancer cell line was obtained from the Chinese Academy of Sciences Cell Bank and cultured in complete Dulbecco’s Modified Eagle Medium (DMEM) supplemented with 5% fetal bovine serum, 100 U/ml streptomycin, and 100 U/ml penicillin at 37 °C in a 5% CO_2 _incubator. All procedures were approved by the Institutional Animal Care and Use Committee (IACUC, protocol number Ek2022113).

### Establishment of a mouse model of liver metastasis from colorectal cancer

CT-26 mouse colon cancer cells were resuspended in DMEM containing 10 g/l agarose. Then, the digested cells were centrifuged at 2000r/min for 5 min. After discarding the supernatant, CT26 cells were diluted by 0.9% saline at a density of 1 × 10⁷ cells/ml. Mice were anesthetized with an intraperitoneal injection of sodium pentobarbital (30 mg/kg) and positioned supine on the operating table. After disinfecting with 1% iodophor, a 1.5 cm incision was made in the left upper abdomen to expose the spleen. A 0.5 ml cell suspension was injected under the splenic capsule. Gentle massage of the bulging capsule facilitated tumor cell entry into the liver via the splenic vein. Once the injection site paleness subsided, the splenic pedicle was ligated, and the spleen was resected to prevent splenic metastasis. After ensuring hemostasis, the abdomen was sutured layer by layer. Post-surgery, mice were transferred to the animal facility for feeding. All surgical procedures adhered to sterile techniques. Before the main experiment, a pre-experiment was conducted to verify the success rate of CLM model mice.

### Experimental protocal

**Figure 1:**
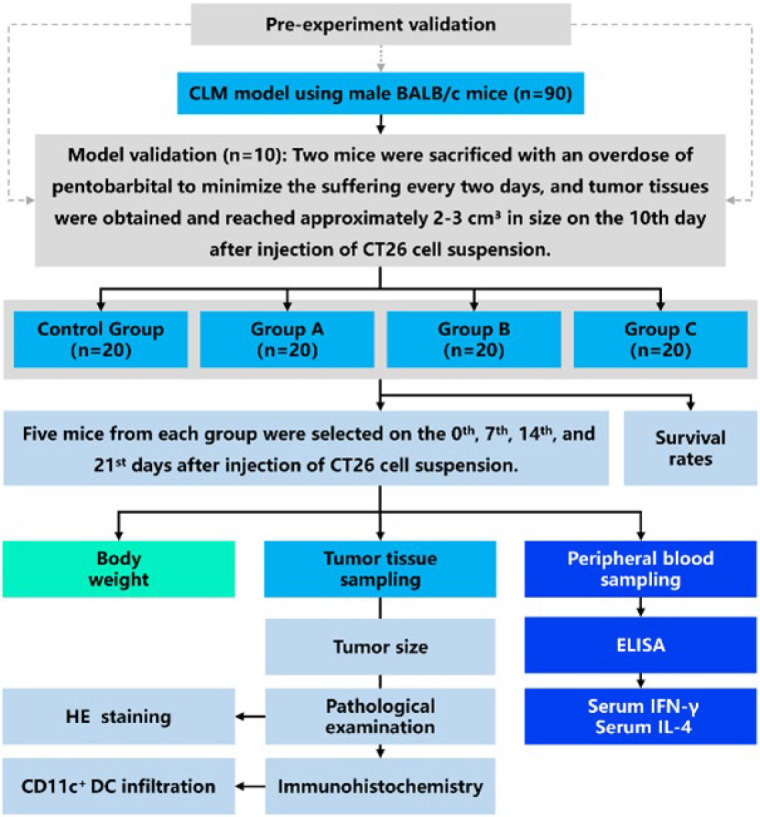
Experimental procedures of study to explore the anti-tumor immune response using a colorectal liver metastases (CLM) mouse model

The flow chart for the procedures of our study is presented in Figure 1. Briefly, to ensure the successful establishment of the CLM mouse model, two mice every two days were randomly selected and sacrificed with an overdose of pentobarbital within 10 days after injection of CT26 cell suspension. As confirmed by laparotomy during pre-experiment validation (20), the remaining 80 mice presenting with tumors approximately 2-3 cm³ in size on the 10^th^ day after injection of CT26 cell suspension were randomly assigned to one of four groups, each group consisting of 20 mice. Group A mice were anesthetized and received an intratumoral injection of mouse GM-CSF (Peprothech, New Jersey, USA) 250 μg/day for three consecutive days using a 17-gauge Trocar needle typically employed for biopsies, followed by inactive cryotherapy. Group B mice, also anesthetized, had a 1.7 mm argon-helium cryoablation probe directly inserted into the tumor tissue. These mice were administered a sham vehicle delivery (250 μg/day of saline for three days). Group C mice were treated with a combination of GM-CSF (250 μg/day for three days) and cryoablation. The Control Group received inactive cryotherapy and an equivalent volume of sham vehicle delivery. The cryoablation procedure was initiated with argon gas at 20% power for 30 sec at a pressure of 17225 kPa (2500 psi). Once the tumor was entirely encapsulated in ice, the core temperature of the tumor tissue could plummet to -120 °C. Subsequently, helium gas was applied to gradually rewarm the tumor to 10 °C. This freezing-thawing cycle was repeated once more using the aforementioned protocol. In Group C, GM-CSF administration was immediately after cryoablation. Peripheral blood and tumor tissue samples were obtained from five mice randomly selected in groups on the 0^th^, 7^th^, 14^th^, and 21^st^ days after injection of CT26 cell suspension. Mouse body weights, tumor sizes, immunohistochemical analysis of the tumors, and serum cytokine levels were subsequently analyzed. For BALB/c mice, euthanasia was considered if they had completely lost their appetite for 24 hr or had exhibited depression accompanied by hypothermia (animals at room temperature below 37 °C). Percent survival was recorded.

### Hemotoxylin and eosin (HE) staining

After euthanasia, tumor specimens were fixed in 10% formaldehyde, embedded in paraffin, and sectioned at 4 μm. Sections were dewaxed in xylene (**2 **× 10 min), rehydrated through graded alcohols (100%, 95%, 85%, 70%, and distilled water; 5 min each), stained with hematoxylin (15 min), differentiated in 1% hydrochloric acid alcohol (30 sec), and counterstained with eosin (5 min). Sections were dehydrated in graded alcohols (70%, 85%, 95%, and 100%; 2 min each), cleared in xylene (10 min), and mounted with neutral resin. Images were captured and analyzed quantitatively using ImageJ software.

### Immunohistochemistry for the DC distribution characteristics

Paraffin-embedded tissue sections (4 μm) were subjected to a two-stage dewaxing process using xylene for 10 min per stage. They were then rehydrated through a graded series of alcohol baths—starting with 100%, followed by 95%, 85%, 70%, and concluding with deionized water—for 5 min in each solution. The sections were treated with a 3% hydrogen peroxide solution at room temperature for 15 min to inactivate endogenous peroxidases. The sections were rinsed with 0.01M phosphate-buffered saline (PBS). They underwent antigen retrieval using a 0.01M buffer to enhance antigen exposure, further facilitated by treatment with pancreatic enzymes. The sections were incubated with a rabbit anti-CD11c primary antibody (at a dilution of 1:150, sourced from Sigma-Aldrich, USA) under 4 °C conditions overnight. On the subsequent day, they were further incubated with a horseradish peroxidase (HRP)-conjugated goat anti-rabbit secondary antibody (supplied by Beijing Zhongxian Jinqiao Biological Co., Ltd, China) for 30 min at a temperature of 37 °C. The immunoreaction was developed using a diaminobenzidine (DAB) chromogen for 5 min (provided by Fuzhou Maixin Biotechnology Co., Ltd, China), which yielded a brown coloration of CD11c^+^ cells. The nuclei were subsequently counterstained with hematoxylin, and the sections were dehydrated through an ascending series of alcohol concentrations, cleared with xylene, and finally mounted with a neutral resin for visualization. CD11c^+^ DC cells were characterized by their distinct membrane staining, which appeared brown. For quantitative analysis, five high-power fields (at ×400 magnification) were selected from each slide, and the CD11c^+^ cells within these fields were manually counted using a light microscope (model CH-2, Olympus, Tokyo, Japan). The infiltration degree of activated DCs within tumor tissues was ascertained by calculating the average count of CD11c^+^ DCs cells from the selected fields across all slides within that group.

### Enzyme-linked immunosorbent assay (ELISA) for serum IFN-γ and IL-4

The contents of serum IFN-γ and IL-4 were measured using commercial ELISA kits (Shanghai Meilian Biotechnology Co., Ltd Shanghai, China), according to the manufacturer’s instructions. Calibrators and samples were placed in front of the 96-well plate. A total of 100 μl calibrators or samples were added into the single micropore of a 96-well plate and incubated for 2 hr at room temperature. Each sample was repeated at least three times. After washing five times, a second polyclonal horseradish peroxidase-labeled antibody was added and then incubated at 37 °C for 60 min. The plate was then washed and added 3, 3′, 5, 5′-tetramethylbenzidine, and incubated at 37 °C in the dark for 25 min. Finally, 100 μl termination solution was added to each micropore, and the plate was incubated for another 30 min. The absorption was measured at 450 nm within 5 min using a microplate photometer. 

### Statistical analysis

Statistical analysis was performed using SPSS 21.0 (SPSS Inc., Chicago, IL, USA). All data were presented as mean ± SD from at least three repeats. The changes in tumor size, the number of DCs, the levels of IFN-γ and IL-4, and the ratio of Th1/Th2 in each group were compared using analysis of variance (ANOVA) followed by LSD. Kaplan Meier log-rank analysis was used to test the survival duration of mice from each group. A *P-*value<0.05 was considered statistically significant.

## Results

### Establishment of a mouse model

**Figure 2:**
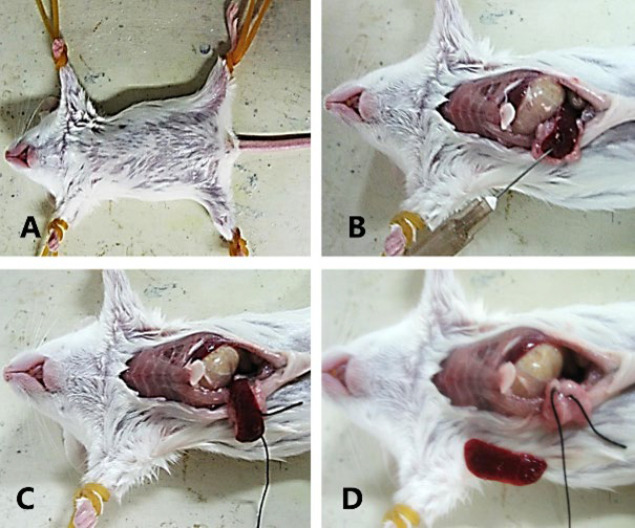
Establishment process of a mouse model of colorectal liver metastases (CLM)

**Figure 3:**
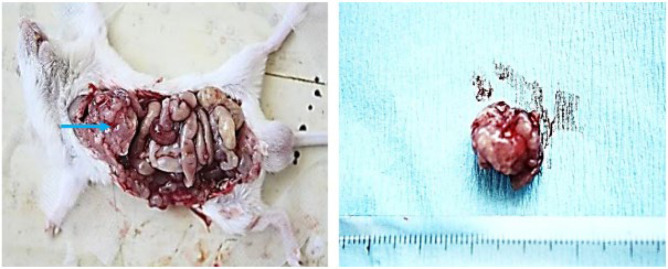
Model validation by exploratory laparotomy and the tumor size with a volume of approximately 2-3 cm³ on the 10th day after injection of CT26 cell suspension

**Figure 4:**
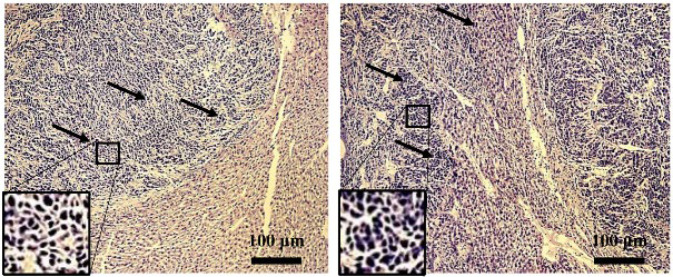
Pathological analysis of colorectal cancer liver metastasis (CLM) sections by hematoxylin and eosin (HE) staining (100×) in male BALB/c mice (6-8 weeks old, weighing 18-22 g).

As shown in Figure 2, a murine model of CLM was successfully established following splenectomy, with a liver metastasis rate of 100% (Figure 2). By the 10^th^ day following the injection of the CT-26 cell lines, numerous metastatic nodules measuring 2-3 cm³ were observed on the liver surface (Figure 3). Histopathological examination revealed cancer cell clusters, accompanied by the formation of cancerous nodules and some spindle-shaped cells. The cancer cells were poorly differentiated and pronounced atypia, presenting with scant cytoplasm, large nuclei with deep staining, and irregular contours (Figure 4).

### Synergistic effect of combined cryoablation and GM-CSF on body weight gain and tumor reduction

**Figure 5:**
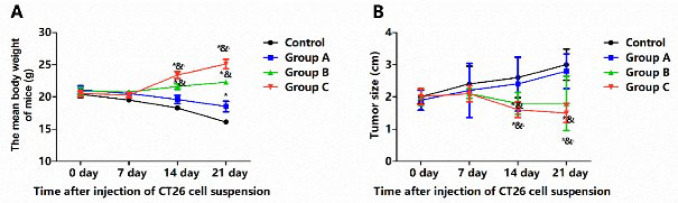
Combined cryoablation and GM-CSF treatment significantly promoted body weight gain and reduced tumor size of male BALB/c mice with colorectal cancer liver metastasis (CLM)

The impacts of combined cryoablation and GM-CSF treatment on body weight (Figure 5A) and tumor size (Figure 5B) were initially assessed. Within the first week post-treatment, none of the interventions significantly inhibited tumor growth, with all groups showing no significant increase in body weight and tumor size by the seventh day after injection of CT26 cell suspension (*P*>0.05). Administration of GM-CSF alone (250 μg/day for three days) in Group A failed to curb tumor progression, which continued to expand without significant deviation from the Control group through the 21^st^ day (*P*>0.05). The mean body weight of mice in both the Control group and Group A continuously decreased. In contrast, cryoablation alone (Group B) or in combination with GM-CSF (Group C) significantly diminished tumor size. It promoted the mean body weight gain after the first week, as compared to the Control group and Group A (*P*<0.05). Notably, Group C exhibited the heaviest weight and the smallest tumor size by the 21^st^ day, compared to Group B (*P*<0.05).

### Enhanced DC infiltration with combined cryoablation and GM-CSF

**Figure 6:**
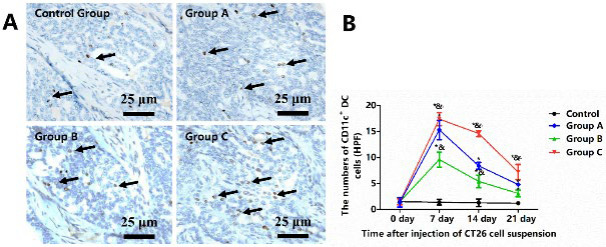
Analysis of activated CD11c+ DCs infiltration within tumor tissue of male BALB/c mice with colorectal cancer liver metastasis (CLM) from Control group, Group A (GM-CSF alone), Group B (cryoablation alone), and Group C (cryoablation + GM-CSF)

The infiltration of activated DCs within tumor tissues was labeled with the primary antibody for anti-CD11c and analyzed via immunohistochemical (IHC) staining (Figure 6A). In the Control group, a sparse distribution of CD11c^+^ DCs was observed. Compared to the Control group, the activated DC counts were increased to a peak on the seventh-day post-treatment in all treatment groups, with the most concentrated CD11c^+^ DCs around the tumor necrosis in Group C (Figure 6B). Thereafter, DC numbers gradually declined until the 21^st^ day. Interestingly, Group B returned to pre-treatment levels of activated DCs (*P*=0.734). Group A and Group C were higher than pre-treatment levels, especially Group C (*P*<0.05). These results indicated that the combined therapy of cryoablation and GM-CSF synergistically increases the number of infiltrating DCs.

### Modulation of serum cytokines by combined cryoablation and GM-CSF

**Figure 7:**
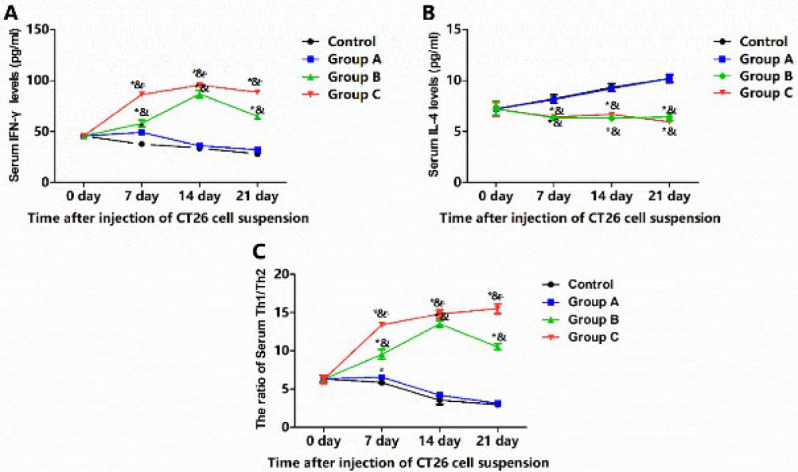
The effect of combined cryoablation and GM-CSF treatment on serum cytokines in male BALB/c mice with colorectal cancer liver metastasis (CLM)

The serum levels of IFN-γ (Figure 7A), IL-4 (Figure 7B), and the Th1/Th2 ratio (IFN-γ/IL-4, Figure 7C) were analyzed. In the Control group, serum IFN-γ levels consistently decreased with the increasing tumor burden. In Group A, GM-CSF significantly boosted serum IFN-γ levels on the seventh-day post-treatment (*P*<0.05), followed by a significant decline below pre-treatment levels (*P*<0.05). By the 14^th^ and 21^st^ days, no significant differences were observed between the Control group and Group A (*P*>0.05). Conversely, cryoablation alone (Group B) or in combination with GM-CSF (Group C) significantly elevated serum IFN-γ levels, reaching a peak on the 14^th^ day and sustaining high levels through the 21^st^ day in both groups. In contrast, serum IL-4 levels in the Control group and Group A progressively increased, peaking on the 21^st^ day, and were significantly higher than those in Group B and Group C on all measured days (*P*<0.05). No significant differences were noted between Group B and Group C at any time point (*P*>0.05). The Th1/Th2 ratio mirrored the pattern observed with IFN-γ levels, with Group C showing the highest ratio on the 21^st^ day. Compared to Group B, Group C exhibited a higher Th1/Th2 ratio on the 7^th^, 14^th^, and 21^st^ days (*P*<0.05). These findings suggest that combined cryoablation and GM-CSF treatment enhances serum IFN-γ levels and the Th1/Th2 ratio while suppressing IL-4 levels.

### Synergistic effect of combined cryoablation and GM-CSF on tumor reduction and survival extension

Kaplan-Meier survival analysis (Table 1) indicated no significant difference in median survival time between Group A (43±2.87 days) and the Control group (42±1.43 days). The longest median survival time was recorded in Group C (72±2.54 days), followed by Group B (55±0.65 days). The mean survival time was significantly extended in Group C (73.2±1.46 days) compared to Group B (53.0±1.27 days) (*P*<0.001), surpassing both the Control group and Group A (*P*<0.001). These findings suggest that combining cryoablation and GM-CSF elicits a synergistic effect in inhibiting tumor growth and extending survival time.

## Discussion

To more closely mimic the conditions of human colorectal cancer post-radical surgery, we successfully established a mouse model of CLM by utilizing a resection method with 80 BALB/c mice. The liver metastasis rate for colorectal cancer reached a striking 100% by splenic injection of CT-26 cell line suspension. Compared to others, this model offers greater stability, straightforward anatomical and surgical characteristics, and a notably high rate of liver metastasis (20). Our findings demonstrated that combining cryoablation with GM-CSF significantly suppressed tumor growth, enhanced immune response by elevating DC counts and the Th1/Th2 ratio, and extended the median survival time in mice bearing tumors.

Our study underscored the synergistic effect of cryoablation and GM-CSF in treating CLM. GM-CSF plays a crucial role in the tumor microenvironment by modulating immune responses through the cytokine network. GM-CSF, a glycoprotein derived from non-hematopoietic sources, could stimulate DCs to present tumor antigens, thus exerting immunomodulatory properties (21). Cryotherapy, as a precise local ablation method, is recognized for preserving a significant amount of healthy liver tissue (22). Although the limited evidence on cryotherapy primarily focuses on prostate cancer, targeted cryoablation of the prostate has demonstrated its effectiveness in cancer treatment (23-25). Our findings demonstrate that the combination of cryoablation with GM-CSF significantly suppressed tumor growth and enhanced immune response by elevating activated DC counts, aligning with the synergistic effect observed in murine glioma models where GM-CSF was found to enhance the immune function of splenic DCs (26). Argon–helium cryoablation has been demonstrated to activate DCs* in vitro* (27) effectively. Our results align with the findings by Xu *et al.* that combined therapy with cryoablation and GM-CSF could synergistically enhance the activation of DCs and induce a robust tumor-specific immunologic response in glioma-bearing mice (26). The synergistic effect between cryoablation and GM-CSF has also been observed in other studies, such as the one by Lin* et al.*, which showed that in-situ administration of dendritic cells following argon-helium cryosurgery enhances specific anti-glioma immunity in mice (28). Moreover, combining cryoablation and GM-CSF resulted in our study’s smallest tumor lesion and the longest mean survival time. Our findings suggest that combining cryoablation and GM-CSF may serve as a potential therapeutic option for CLM treatment. Exploring cryo-immunological responses will provide a rationale and experimental foundation for developing synthetic therapies for advanced cancer.

A growing body of evidence suggested that cytokines and chemokines played a crucial role in the progression of colorectal cancer by modulating immune suppression and the tumor microenvironment (29, 30). In our study, the combination with GM-CSF notably enhanced serum IFN-γ levels and the Th1/Th2 ratio, which correlated with the post-treatment changes in DC counts and, inversely, serum IL-4 levels. Recent progress in GM-CSF-based cancer immunotherapy has been reviewed, highlighting strategies such as GM-CSF monotherapy, GM-CSF-secreting cancer cell vaccines, and GM-CSF combination therapy, which contribute to the regulation of immunosuppression in the tumor microenvironment (31). GM-CSF can convert DCs in cancers to the pro-inflammatory phenotype with better antigen-presentation capabilities to cytotoxic T cells or to the tolerogenic phenotype that suppresses cytotoxic T cells at the expense of regulatory cells (32). Furthermore, a study found that cryoablation induces greater inflammatory and coagulative responses than radiofrequency ablation or laser-induced thermotherapy in a rat liver model, suggesting a potential synergy with immunotherapeutic approaches (33, 34). In a pilot study, percutaneous cryoablation with aerosolized GM-CSF enhanced the secretion of Th1 cytokines, induced tumor-specific cytotoxic T lymphocytes, and antitumor antibody responses in renal cell cancer with lung metastasis, which also supported our conclusion (35). Similarly, in a recent study involving a small cohort of 20 patients, the combination of GM-CSF with cryotherapy was found to bolster the immune response in prostate cancer treatment (25). These studies support our observation that cryoablation and GM-CSF can promote the reversal of antitumor immunity towards Th1 immune polarization, providing a significant advantage within the body. Our findings suggest that combining cryoablation with GM-CSF activates DCs and amplifies the immune response to improve immunosuppression in the tumor microenvironment.

Several limitations in our study should be issued. First, a CLM mouse model was used, which might not fully reflect clinical reality. Future studies with larger and longer clinical trials are needed to confirm our findings in CLM patients. Second, we mainly measured the antitumor response by counting DCs and checking serum cytokines (IL-4 and IFN-γ) with immunohistochemistry and ELISA. Third, future studies should use flow cytometry to understand better changes in CD8+ T cells in and around tumors and the blood. This would help us see how cryoablation with GM-CSF treatment affects the immune system against tumors. Lastly, the tumor environment is complex, involving many signals and immune-suppressing factors. More research is needed to explore how cryoimmunology works with combined therapies, like how macrophages and GM-CSF can help prevent tumor recurrence and spread.

Arrows indicate the neoplastic cells with marked pleomorphism

A) Changes in body weight after injection of CT26 cell suspension. B) Changes in tumor size after injection of CT26 cell suspension. vs Control group, *P<0.05; vs Group A, & *P*<0.05; vs Group B,*P*<0.05 on the 7^th^ day, 14^th^ day, 21^st^ day after injection of CT26 cell suspension, respectively.

A) Tumor tissue sections were labeled with the primary antibody for anti-CD11c by immunohistochemical (IHC) staining under a high power field (×400). Arrows indicate the activated CD11c+ DC cells stained brown. B) Numbers of CD11c+ DC cells. vs Control group, *P<0.05; vs Group A, &P<0.05; vs Group B, P<0.05 on the 7th day, 14th day, 21st day after injection of CT26 cell suspension, respectively.

**Table 1:** Analysis of survival time in model BALB/c mice after injection of CT26 cell suspension

Groups	Mean survival time (day)	Median survival time (day)
Control	40.4±1.32	42±1.43
Group A	43.1±1.53	43±2.87
Group B	53.0±1.27^*^	55±0.65
Group C	73.2±1.46^*^	72±2.54

Log-Rank analysis: *Compared with Control group (*P*<0.001); Compared with group B (*P*<0.001).

## Conclusion

Our findings have expanded the evidence of cryoablation in the treatment of CLM. Combining cryoablation with GM-CSF exerted a potent antitumor effect, enhancing the number of DCs within the tumor microenvironment, bolstering the antitumor immune response, and extending the median survival time in mice bearing tumors. More clinical studies are needed to improve cryotherapy and maximize its benefits for patients with CLM. 

## Declaration

We have not used any AI tools or technologies to prepare this manuscript.
